# Evidence for seasonal migration by a cryptic top predator of the deep sea

**DOI:** 10.1186/s40462-024-00500-x

**Published:** 2024-09-24

**Authors:** William K. Oestreich, Kelly J. Benoit-Bird, Briana Abrahms, Tetyana Margolina, John E. Joseph, Yanwu Zhang, Carlos A. Rueda, John P. Ryan

**Affiliations:** 1 Monterey Bay Aquarium Research Institute, Moss Landing, CA USA; 2 Center for Ecosystem Sentinels, Department of Biology, University of Washington, Seattle, WA USA; 3 Naval Postgraduate School, Monterey, CA USA

**Keywords:** Deep sea, Movement ecology, Migration, Resource tracking, Phenology, Bioacoustics, Sperm whale (*Physeter macrocephalus*), Echolocation, Marine megafauna, Pelagic

## Abstract

**Background:**

In ecosystems influenced by strong seasonal variation in insolation, the fitness of diverse taxa depends on seasonal movements to track resources along latitudinal or elevational gradients. Deep pelagic ecosystems, where sunlight is extremely limited, represent Earth’s largest habitable space and yet ecosystem phenology and effective animal movement strategies in these systems are little understood. Sperm whales (*Physeter macrocephalus*) provide a valuable acoustic window into this world: the echolocation clicks they produce while foraging in the deep sea are the loudest known biological sounds on Earth and convey detailed information about their behavior.

**Methods:**

We analyze seven years of continuous passive acoustic observations from the Central California Current System, using automated methods to identify both presence and demographic information from sperm whale echolocation clicks. By integrating empirical results with individual-level movement simulations, we test hypotheses about the movement strategies underlying sperm whales’ long-distance movements in the Northeast Pacific.

**Results:**

We detect foraging sperm whales of all demographic groups year-round in the Central California Current System, but also identify significant seasonality in frequency of presence. Among several previously hypothesized movement strategies for this population, empirical acoustic observations most closely match simulated results from a population undertaking a “seasonal resource-tracking migration”, in which individuals move to track moderate seasonal-latitudinal variation in resource availability.

**Discussion:**

Our findings provide evidence for seasonal movements in this cryptic top predator of the deep sea. We posit that these seasonal movements are likely driven by tracking of deep-sea resources, based on several lines of evidence: (1) seasonal-latitudinal patterns in foraging sperm whale detection across the Northeast Pacific; (2) lack of demographic variation in seasonality of presence; and (3) the match between simulations of seasonal resource-tracking migration and empirical results. We show that sperm whales likely track oceanographic seasonality in a manner similar to many surface ocean predators, but with dampened seasonal-latitudinal movement patterns. These findings shed light on the drivers of sperm whales’ long-distance movements and the shrouded phenology of the deep-sea ecosystems in which they forage.

**Supplementary Information:**

The online version contains supplementary material available at 10.1186/s40462-024-00500-x.

## Background

The movement strategies that animals use to track resources in space and time drive many aspects of their ecology, mediate their ability to respond to environmental perturbations, and provide insight into the spatiotemporal dynamics of the ecosystems they inhabit [1]. These individual and group-level movement strategies typically result from spatiotemporal patterns of resource availability [2], and manifest in distinct patterns of population-level distribution in space and time [3]. For example, nomadic resource tracking has evolved in aseasonal and unpredictable environments, leading to irregular patterns of individual movement and population distribution [4]. Conversely, many species inhabiting seasonal ecosystems have evolved to undertake seasonal migrations between distinct ranges [4] or perform partial migrations, whereby a specific demographic of the population undertakes migration [5]. These seasonal migrations between distinct habitats (sometimes referred to as “to-and-fro” migrations), as in the migrations of many baleen whales, are distinguished by their persistent, relatively direct movements undistracted by proximate resources [6]. Other seasonal migrants (e.g., many ungulates) undertake seasonal movements to track the phenology of proximate resources (e.g., forage, favorable abiotic conditions, etc.) en route as resource availability propagates across spatiotemporal gradients such as latitudes or elevations [7, 8]. These resource-tracking migrations have recently gained attention as an important connection between ecosystem dynamics and animal movement, closely linking ecosystem phenology with that of seasonal animal migrations [1, 9]. Such resource tracking has been shown to provide a number of individual and population-level benefits, from enabling animals to have more prolonged access to food [10], to increasing fat gain [11] and allowing migratory populations to have higher growth rates than sedentary populations [12]. These linkages between resource dynamics and animal movement strategies are increasingly well-understood in seasonal terrestrial [2, 7, 9, 13], freshwater [14], coastal marine [15], and epipelagic [16–21] ecosystems across the globe.

Few studies have assessed these connections between ecosystem dynamics and animal movement in Earth’s largest habitable space: deep pelagic ecosystems. These oceanic waters deeper than 200 m, where little sunlight penetrates, have historically been characterized as stable and aseasonal but are poorly understood [22]. However, a growing body of evidence suggests elements of seasonality in the deep sea. For example, oceanographic studies have documented seasonal variation in the transport of biomass from the surface to the deep [23–25]. Further research has documented seasonality in sightings and biomass of low and mid-trophic level organisms in the mesopelagic [26–28]. Yet understanding of deep-sea phenology remains limited, particularly for highly mobile and high-trophic-level animals. This knowledge gap is underpinned by the challenge of making continuous and detailed observations in these ecosystems [22]. Given the global extent, high endemic biodiversity, and major role in global biogeochemical cycles of deep pelagic ecosystems, understanding the phenology of these ecosystems and the evolved movement strategies of their inhabitants is important to advance fundamental ecology and inform ecosystem management.

We address this gap by integrating long-term passive acoustic monitoring data and movement simulations for a deep pelagic top predator, the sperm whale (*Physeter macrocephalus*). Sperm whales are a deep-diving oceanic predator, diving to depths of hundreds to thousands of meters [29] to forage on diverse deep pelagic prey [30]. Thus, studying the movement patterns of these ocean giants can provide a rare window into the phenology of the deep-sea environment. In addition, sperm whales produce the loudest known biological sounds [31] which not only reveal the presence of this often-cryptic species over large ocean volumes, but also transmit rich behavioral and demographic information about detected individuals. Echolocation clicks are central to the foraging ecology of sperm whales in the low-light conditions of the deep sea, and further indicate individuals’ behavioral state (foraging), size (both inter-click-interval [32] and inter-pulse-interval within individual clicks [33] correlate with size), and sex and age-class (sperm whales are sexually dimorphic [34], allowing for sex and age-class identification via inter-click-interval [32]). Sperm whales use echolocation in both the meso- and bathypelagic [35] to locate a variety of squid and fish prey species [30]. Because of this essential foraging function, sperm whales produce echolocation clicks year-round and at all hours of the day. As a result, patterns of sperm whale echolocation click detection can provide insight into the phenology of both this top predator and the deep pelagic ecosystems in which they forage.

**Fig. 1:**
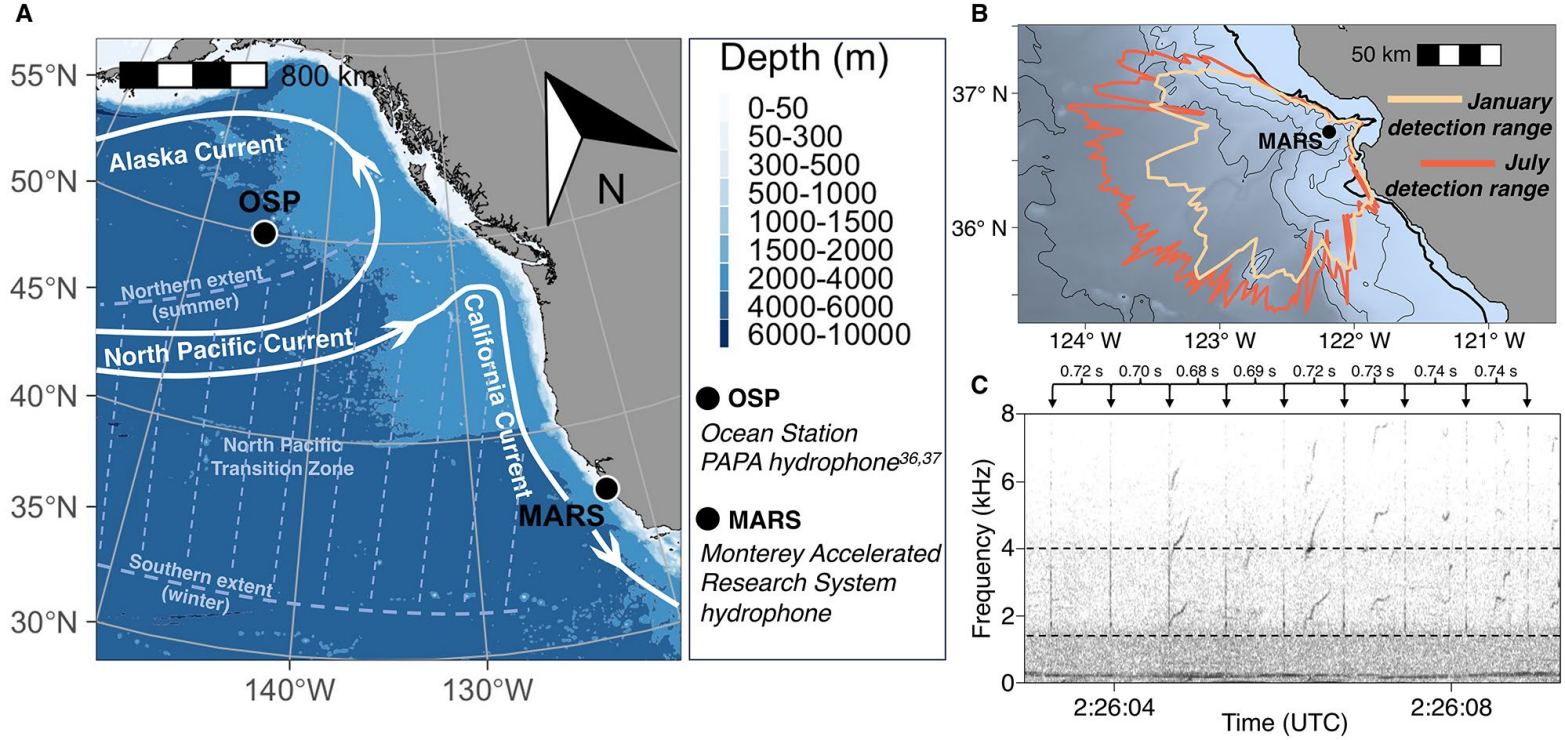
Study system and acoustic methods. (**A**) The Northeast Pacific Ocean, showing the location of passive acoustic recordings from the present study (Monterey Accelerated Research System (MARS) in the Central California Current System) and previous studies [36, 37] (Ocean Station PAPA (OSP) in the Gulf of Alaska). (**B**) The Central California Current System, indicating winter and summer detection ranges for sperm whale echolocation clicks produced at 500 m depth (see Methods and SI for additional depths) based on average January and July oceanographic conditions over the period 2016–2022. The circle indicates MARS (891 m depth), with contours representing the 200 m isobath (thicker line) and multiples of 1000 m (thinner lines). (**C**) Example spectrogram of audio recorded at MARS on November 30, 2022, showing a period when a single foraging sperm whale’s echolocation clicks (impulsive, broadband signals) were clearly visible and audible. Dashed horizontal lines indicate the minimum and maximum frequencies of the automated energy detector used to detect sperm whale echolocation clicks. Note the near-constant inter-click-interval used to discern echolocating sperm whales from other impulsive sound sources in this frequency range

In the Northeast Pacific, foraging sperm whales have been detected acoustically year-round, specifically in the Gulf of Alaska (GoA) [36–38]. Individuals of this population have expansive home ranges, exhibiting wide-ranging movements which include travel between the GoA and the Central California Current System (CCCS; Fig. 1A) among other lower-latitude habitats [39–41]. Yet the regularity, seasonality, and behavioral context of such movements have historically remained unclear. Previous studies based on individual-level sightings, genetic, and limited telemetry data have hypothesized that latitudinal movements are likely irregular, resulting from aseasonal nomadic movements [40] consistent with the canonical view of aseasonal deep-sea ecosystems. Yet recent acoustic studies in the GoA have suggested seasonality in foraging sperm whales’ presence [36–38], challenging the hypothesis of aseasonal nomadic movements. Others have suggested that long-distance latitudinal movements represent migration between distinct high-latitude foraging and low-latitude breeding habitats [42], akin to the seasonal migrations of many baleen whales. Sex-specific partial seasonal migration (with only adult males undertaking migration to higher latitudes) has also been hypothesized based on individual-level sightings data [34, 43], but females have also been observed in both the GoA [40] and CCCS [40, 44]. Further, individuals with small body size (females and juveniles) are heard year-round in the GoA [38], counter to the hypothesis that exclusively adult males undertake long-distance movements to high latitudes. While individual-level telemetry data can often provide sufficient sample sizes to understand population-level seasonal movement strategies [16], individual tracks of sufficient duration to assess seasonal movement are extremely limited for this sperm whale population [39]. As with most inhabitants of deep pelagic ecosystems, this murky understanding of sperm whales’ movement strategies arises from the challenge of observing their behavior persistently at sufficient scale [45, 46] and limited understanding of phenology in their foraging habitat.

Here, we investigate the strategies underlying movements of this deep pelagic top predator in the Northeast Pacific. We consider four hypothesized movement strategies. Three have previously been hypothesized: nomadic resource tracking [40], seasonal to-and-fro migration between distinct habitats [39, 42], and sex-specific partial seasonal migration [34, 42], The fourth, seasonal resource-tracking migration akin to that observed in many surface ocean and terrestrial predators [16, 19], is hypothesized here based on growing evidence of seasonality in the deep sea at lower trophic levels [23–28]. We first characterize seasonal patterns of foraging sperm whale presence in the Central California Current System as compared to previously published results from the Gulf of Alaska by applying automated acoustic detection methods to more than seven years of passive acoustic recordings. Passive acoustic monitoring approaches provide a valuable Eulerian lens to assess population-level animal presence and behavior [47], particularly in largely inaccessible oceanic ecosystems when Lagrangian tracking data (e.g., telemetry) is scarce (as with sperm whales in the Northeast Pacific), and in cases where information beyond presence alone (e.g., behavioral state) can be discerned from the properties of detected acoustic signals [47, 48, 49]. We then test the alternative hypotheses by comparing these empirical patterns with emergent patterns derived from simulations of individual-level movement driven by each of the hypothesized movement strategies. Finally, we compare empirically observed seasonal-latitudinal patterns of foraging sperm whale presence to seasonal-latitudinal patterns in the location of the North Pacific Transition Zone, the dominant foraging habitat which numerous surface ocean predators track in the North Pacific [16, 50]. Hypothesis-testing using this integrated approach allows us to *(i)* determine the unknown seasonality and regularity of foraging sperm whale presence in the Central California Current System and *(ii)* evaluate the individual-level strategies underlying sperm whales’ wide-ranging movements by comparing simulated and observed patterns.

## Methods

### Hydrophone recordings

To assess seasonal and interannual patterns of sperm whale presence in the CCCS, we analyzed passive acoustic recordings between 2015 and 2022 with nearly continuous (> 95%) temporal coverage. Acoustic recordings were collected on the Monterey Accelerated Research System (MARS) cabled observatory (36° 42.75’N, 122° 11.21’W; depth 891 m; Fig. 1A), located on the continental slope outside Monterey Bay, CA. The hydrophone, which sits 1 m above the seafloor, is an Ocean Sonics icListen HF digital hydrophone with a bit depth of 24, digital sensitivity of -40 dB, voltage sensitivity of -169 dBV re µPa, and a dynamic range (1.0 Hz bandwidth) of 148 dB. The original hydrophone was deployed in July 2015 and was replaced by a new instrument of the same model in June 2017. All recording maintained a sample rate of 256 kHz. Manufacturer-measured calibrations for each hydrophone were applied after data collection. All recordings were decimated [51] to a sample rate of 16 kHz before analysis. While directional components of sperm whale echolocation clicks can have a peak frequency exceeding the Nyquist frequency of these 16 kHz audio files [31], this sample rate allows for reliable detection of the omnidirectional low-frequency component of these clicks. Previously, these clicks have been reliably detected in audio files with a sample rate as low as 1 kHz [36].

### Passive acoustic analyses

Sperm whales produce a variety of click types associated with distinct behaviors. The present analysis focused only on “usual” clicks, which are used for echolocation [34] and are hereafter referred to as clicks. We used a two-step automated workflow (detection and filtration) to determine presence or absence of sperm whale clicks at daily resolution.

Candidate detections of individual clicks were generated using a band limited energy detection (BLED) approach implemented in Raven Pro v1.6 [52]. We manually tuned the parameters of a BLED (Table S2) to maximize the chances of detecting sperm whale clicks under a range of background noise scenarios, but this first step in acoustic processing also generated many false positives. These false positives were filtered out in the second step of our automated workflow by searching BLED results for repetitive, evenly-spaced sequences of detections matching the known inter-click interval (ICI) range of sperm whale clicks (~ 0.5–2.0 s [53]). Because the intervals between clicks in sperm whale echolocation sequences are largely regular but not exactly constant (Fig. 1C), we calculated the time difference between each BLED detection (inter-detection interval; IDI), then rounded to the nearest quarter second to enable a search for sequences of detections with a near-constant IDI. Each day of recording was automatically searched for IDI sequences matching three criteria: (1) rounded IDI must be between 0.5 and 2.0 s (inclusive); (2) rounded IDI must be constant; and (3) the number of consecutive IDI values meeting criteria (1) and (2) must meet a sufficient number of repetitions (r) to confidently determine sperm whale echolocation click presence. We considered any day with at least one sequence meeting these criteria to have sperm whale clicks present; all other days were considered to have such clicks absent. Setting the number of repetitions required to consider clicks present can significantly impact the performance of this automated workflow at daily resolution (Figure S1; Table S2). The optimal value for this parameter was determined via comparison to manual identification of sperm whale search clicks. Manual assessments were completed for one randomly chosen day of each month in, 2016, 2018, 2020, and 2022, as well as two days of known sperm whale presence near MARS in late 2022. These 50 days provided a representative range of soundscape conditions by covering the full seasonal cycle, including periods recorded by each of the two consecutively-deployed hydrophones, and including recording periods before (2016, 2018), during (2020), and following (2022) the COVID-19 pandemic and its associated changes in anthropogenic noise conditions in the region [54]. We found optimal performance at *r* = 6, yielding a daily balanced accuracy of 96% (precision = 96%, recall = 96%) and false positive rate of 4% (Figure S1; Table S2).

Using this time series of daily-resolution presence and absence, we then calculated monthly percent of recording days with foraging sperm whales present over the time series. This metric is effective in the study context for multiple reasons: (1) it provides sufficient temporal resolution to assess seasonal trends, the primary timescale of focus in this study; (2) automated detector performance is very high at daily resolution (Figure S1), providing high confidence in this metric; and (3) this metric matches that used in previous studies of foraging sperm whale presence at Ocean Station PAPA in the Gulf of Alaska (GoA) over the years 1999–2001 [36] and 2007–2012 [37], allowing for comparison of seasonal presence of foraging whales across a large latitudinal range. Monthly percent presence values from the GoA were determined by digitizing the figures presenting this information in previous studies [36, 37] and were later used in comparison to simulation results. The seasonal patterns from these earlier studies [36, 37] match those recorded more recently in the GoA [38] (2011–2019), with all studies showing a summer maximum and winter minimum of foraging sperm whale presence in the GoA.

Seasonality in the detection of foraging sperm whales in the CCCS was assessed statistically via a generalized additive model of monthly percent presence as a function of month with year nested as a random effect, to test for the deviance in percent presence explained by the seasonal cycle alone. Finally, because inter-click-interval (ICI) correlates with body size and demographic group [32] and therefore can help assess the hypothesis of sex-specific partial migration, we calculated the ICI of all detected click sequences in the time series. The automated detector used here relies on near-constant ICI; therefore our analyses exclude transitionary periods into prey-capture creaks which could inaccurately skew toward smaller ICI values. As part of the manual validation process described above for acoustic presence vs. absence, we also manually confirmed the presence of individuals across ICI-determined size classes throughout the full annual cycle. We used ANOVA to test for seasonal effects on natural-log-transformed ICI distribution. To test for correlation between monthly mean ICI and monthly foraging sperm whale presence, we used linear regression.

### Estimation of detection range

Because seasonality in foraging sperm whale detection could be influenced by seasonal differences in detection range, we assessed seasonality in both ambient noise levels and acoustic propagation loss between sound source and the acoustic receiver at MARS. From daily files of 16 kHz audio data spanning the full study period, daily mean noise levels (single-sided mean-square sound pressure spectral density) were computed for the frequency band targeted by the click detector (1.4–4 kHz). These daily ambient noise values were binned by month across years to examine seasonality.

Acoustic propagation loss was modeled for January and July to assess seasonality in click detection range (Fig. 1B). We modeled acoustic transmission loss for an impulsive sound source at 2.7 kHz (the center frequency of the BLED), 185 dB re: 1µPa at 1 m (peak level of the omnidirectional low-frequency component of sperm whale echolocation clicks [55]), and source depths of 100, 500 and 1000 m (typical of echolocation in foraging sperm whales in many ecosystems [29, 35, 56]), received at the location of MARS. Range-dependent sound speed profiles for the January and July model runs were calculated from the climatological mean of seawater temperature and salinity over the period 2016–2022 as estimated by the HYCOM (HYbrid Coordinate Ocean Model) data assimilative system [57] with 4.8-minute spatial resolution. Acoustic propagation loss was then calculated for each of 360 1° bearings from MARS (Fig. 1B) using a wave-theory parabolic equation model that accounts for absorption in both the water column and the bottom, scattering in the water column and at the surface and bottom, geometric spreading (spherical and cylindrical), refraction, and diffraction [58]. This acoustic propagation modeling specifically considers the region’s bathymetry, sediments and corresponding geoacoustic parameters, and surface winds [59]. Finally, detection range for each source depth and season was estimated for each of these 360 bearings, requiring received level at MARS to exceed 5.0 dB (SNR of the click detector, Table S3) above monthly median ambient noise levels (Figure S3).

### Simulation of individual-level movement strategies

**Fig. 2:**
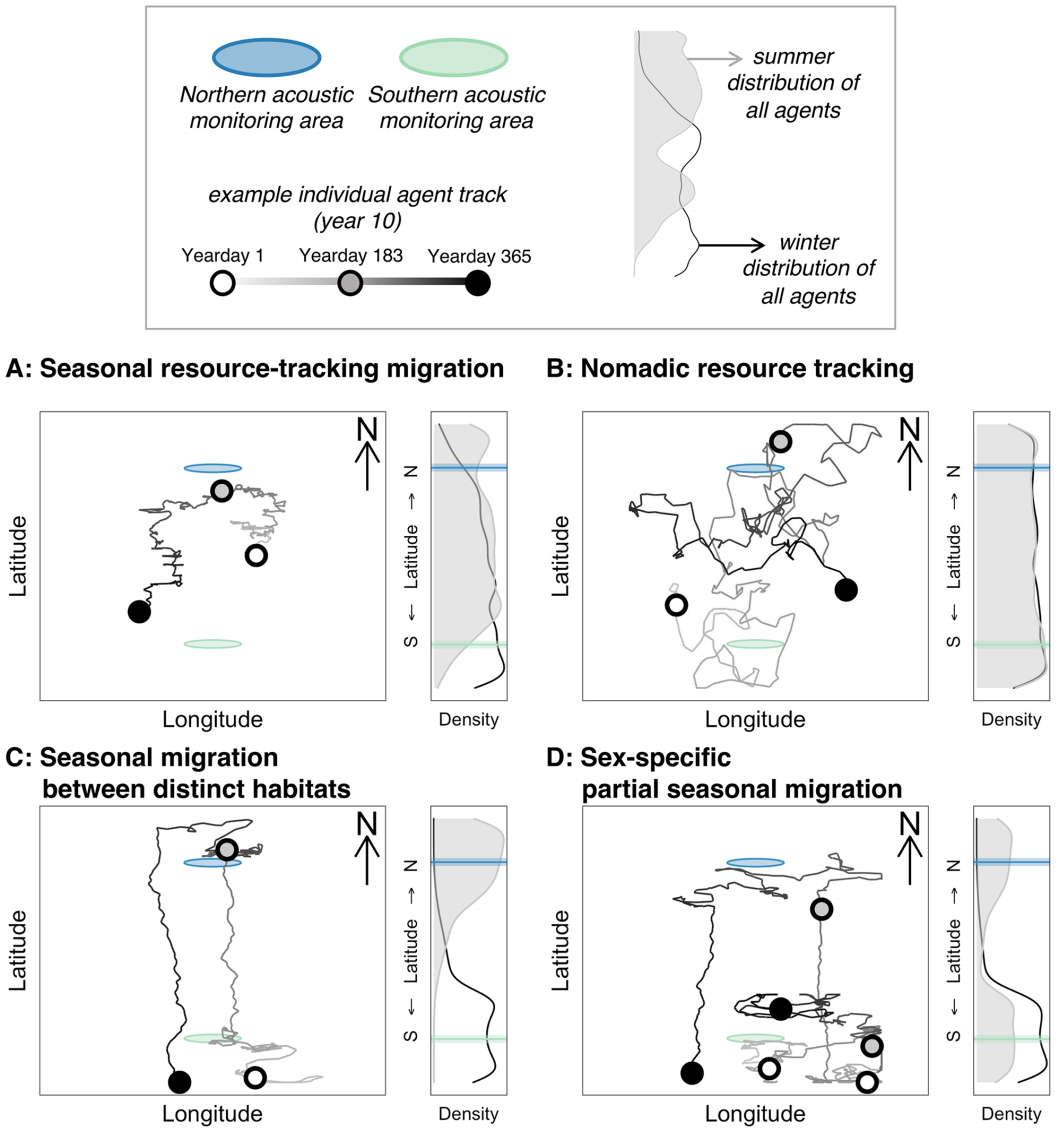
Simulated individual-level movement strategies. Top panel provides a legend for the simulation domain. In each of the panels **A**-**D**, one individual’s track (two individuals, one female and one male, in the case of sex-specific partial seasonal migration) is shown from year 10 of the simulation alongside the summer and winter distribution of all individuals over years 2–10. Circular acoustic monitoring areas appear elliptical due to distortion of the simulation domain in this visualization to highlight individual track details

To test hypotheses regarding the individual-level movement strategies underlying empirically observed patterns of foraging sperm whale presence, we developed individual-based movement simulations which we compared to empirical patterns of whale detection. We employed simulations in which agents move through a spatial domain with two hydrophone “listening ranges” (one at higher latitude and one at lower latitude), analogous to passive acoustic monitoring of sperm whales in the GoA [36, 37] and the CCCS (present study). In all simulations, 100 agents moved daily according to strategy-specific decisions over a ten-year period. The spatial domain in which these simulations occurred is not meant to specifically represent the spatial dimensions of the North Pacific or hydrophone listening ranges used in the present or previous studies. Instead, this spatial domain (described in greater detail in the Supporting Information) provides a simplified arena for testing realistic individual movement strategies [60] and their influence on population-level spatiotemporal patterns of acoustic detection (Fig. 2).

We used empirically determined information about step length and turn angle distributions, as well as seasonality of movement, for well documented movement strategies across diverse taxa and ecosystems [60] to formulate movement decision rules for agents representing the four hypothesized movement strategies (Table S3). We examined the population-level acoustic detection patterns resulting from each of these four movement strategies via four separate simulations with agents subject to these decision rules. At each daily timestep of each ten-year simulation, we recorded each agent’s position and presence or absence in each of the simulated hydrophone listening ranges. The population-level patterns resulting from each simulation were compared to empirical observations of foraging sperm whale seasonality in the GoA [36, 37] and the CCCS (present study) by calculating the root-mean-square deviation of simulated monthly mean acoustic detection results from both listening ranges relative to empirical results. For a complete description of simulation parameters (following methods established by [60]), see the Supporting Information and code [61] accompanying this manuscript.

### Comparison to oceanographic seasonality

To consider whether presence of foraging sperm whales tracks seasonality in oceanographic habitat in a manner similar to many surface ocean predators [16], we compared seasonal patterns of foraging sperm whale presence to seasonal patterns in the location of the North Pacific Transition Zone (NPTZ; Fig. 1A). The NPTZ is a major oceanographic feature in the North Pacific Ocean, representing a transition in surface primary productivity between the subpolar and subtropical gyre [62] and serving as important foraging habitat for a wide range of predators in the surface ocean [16, 50]. The latitudinal position of the NPTZ varies seasonally, reaching its southern extent in the winter and northern extent in the summer (Fig. 1A; [62]). We calculated the monthly latitude of the NPTZ for each month of the acoustic time series as in [62], identifying the mean latitude of the 18 °C sea surface temperature (SST) isotherm between 160 and 180 °W using monthly composite Aqua MODIS 0.025° daytime SST imagery (for comparison to 2015–2022 CCCS acoustic metrics) and Pathfinder v5.3 0.0417° daytime SST imagery (for comparison to pre-2006 GoA acoustic metrics and to fill Aqua MODIS data gaps). We then compared the monthly percent of days with foraging sperm whale present to the monthly NPTZ latitude via model II (ranged major axis) linear regression, given uncertainty in both the independent and response variables.

### Software

All analyses of click detections and individual-level movement simulations were conducted in R v4.2.0 [63]. The maps in Fig. 1 were created using the packages “ggOceanMaps” [64], “geosphere” [65], and “marmap” [66]. Background noise, acoustic propagation, and satellite-based oceanographic analyses were conducted in Matlab [67]. Candidate click detections were generated using Raven Pro v1.6 [52].

## Results

### Seasonality in acoustic detection

**Fig. 3:**
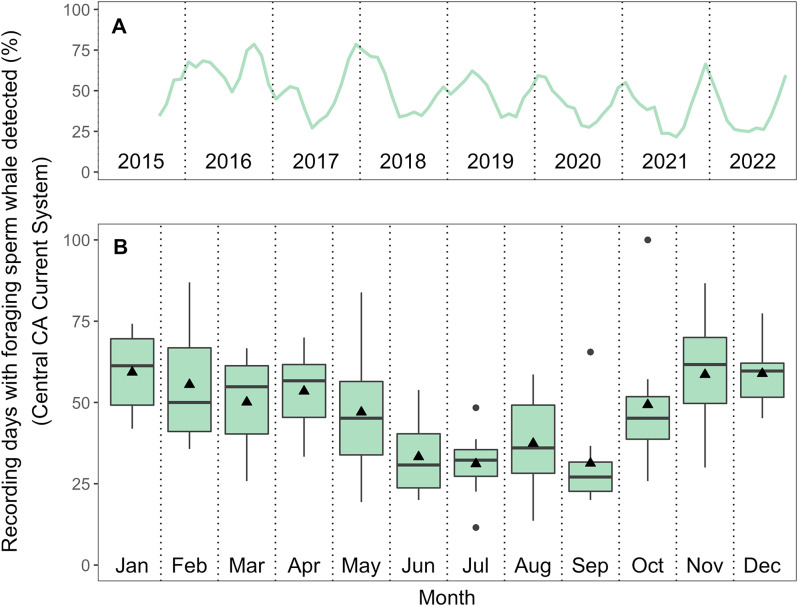
Empirically observed foraging sperm whale presence in the Central California Current System. (**A**) Monthly percent presence over the full study period (smoothed with a 3-month running mean). (**B**) Annual cycle of echolocating sperm whale presence over the full study period (Aug 2015 – Dec 2022). Boxplots show the median (center line), mean (triangle), 25th -75th percentile (box), ± 1.5*IQR (whiskers), and outlying points. A generalized additive model (GAM) revealed a significant relationship between monthly percent of days with presence and month, with year nested as a random effect (*p* < 0.001; 45.4% deviance explained; Figure S2)

Acoustic detection revealed year-round, seasonally varying presence of foraging sperm whales in the Central California Current System (CCCS; Fig. 3). The frequency of foraging sperm whale presence in the average annual cycle reached a maximum in January (mean of 59.3% of days present) and a minimum in July (mean of 31.1% of days present). A generalized additive model revealed a significant relationship between monthly percent of days with presence and month, with year nested as a random effect (*p* < 0.001; 45.4% deviance explained; Figure S2), indicating seasonality in foraging sperm whale presence in the CCCS. Detection seasonality did not result from seasonal changes in ambient noise or maximum detection range. Maximum click detection range was slightly greater during the summer minimum in click detections relative to detection range during the winter detection maximum (Fig. 1B, S3), indicating that the degree of seasonality shown here (Fig. 3B) is a conservative estimate. Interannually, the percent of recording days on which foraging sperm whales were detected varied little, with the exception of 2016 (Fig. 3A). Foraging sperm whales were detected on 63.4% of recording days in 2016, whereas the percentage in all other years varied between 38.6 and 49.9%. These daily detection estimates are potentially conservative given that only the lower-frequency components of sperm whale echolocation clicks are considered here.

### Seasonality of acoustically detected demographic groups

**Fig. 4:**
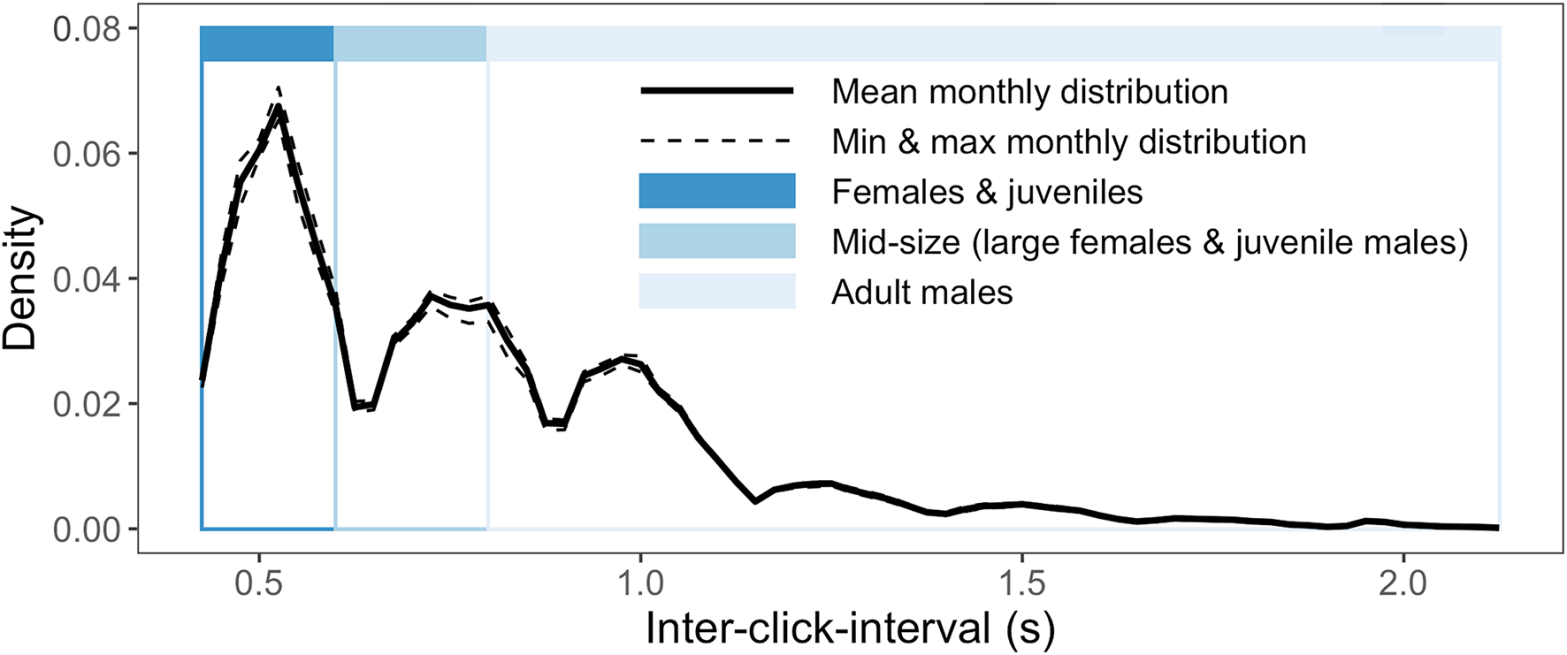
Inter-click-interval (ICI) monthly distributions (relative density). Solid line represents the mean monthly distribution of ICI for detected sperm whale echolocation click sequences over the full study period. Dashed lines represent the minimum and maximum monthly ICI distributions at each ICI value. Colors indicate the demographic groups associated with ICI values as per [32, 38]

Inter-click-interval (ICI) can be used as a proxy for body-size and therefore demographics of acoustically detected individuals in this sexually dimorphic population [32]. Similar to acoustic results from the GoA [38], we detected three clear modes of ICI in automatically-detected click sequences (Fig. 4). It is important to note that this approach does not account for re-sampling of the same individual, meaning that the resulting click sequence ICI data are most appropriate simply for assessing seasonality in the *presence of any individuals* within specific demographic groups (i.e., assessment of the *abundance of individuals* within specific demographic groups is not appropriate in this analysis). We found no seasonality or interannual variation in the distribution of detected ICIs (and therefore, demographics): ANOVA on natural-log-transformed ICI data indicated no significant relationship between month (F = 1.52, df = 11,70, *p* > 0.1) or year (F = 1.70, df = 7,70, *p* > 0.1) and ICI. We detected individuals with both large body size (adult males, ICI > 0.8 s [32, 38]) and small body size (females and juveniles, ICI < 0.6 s [32, 38]) in every individual month of the seven-plus year study period. We also find no relationship between monthly mean ICI and monthly percent presence (Figure S4).

### Individual-level movement simulations

**Fig. 5:**
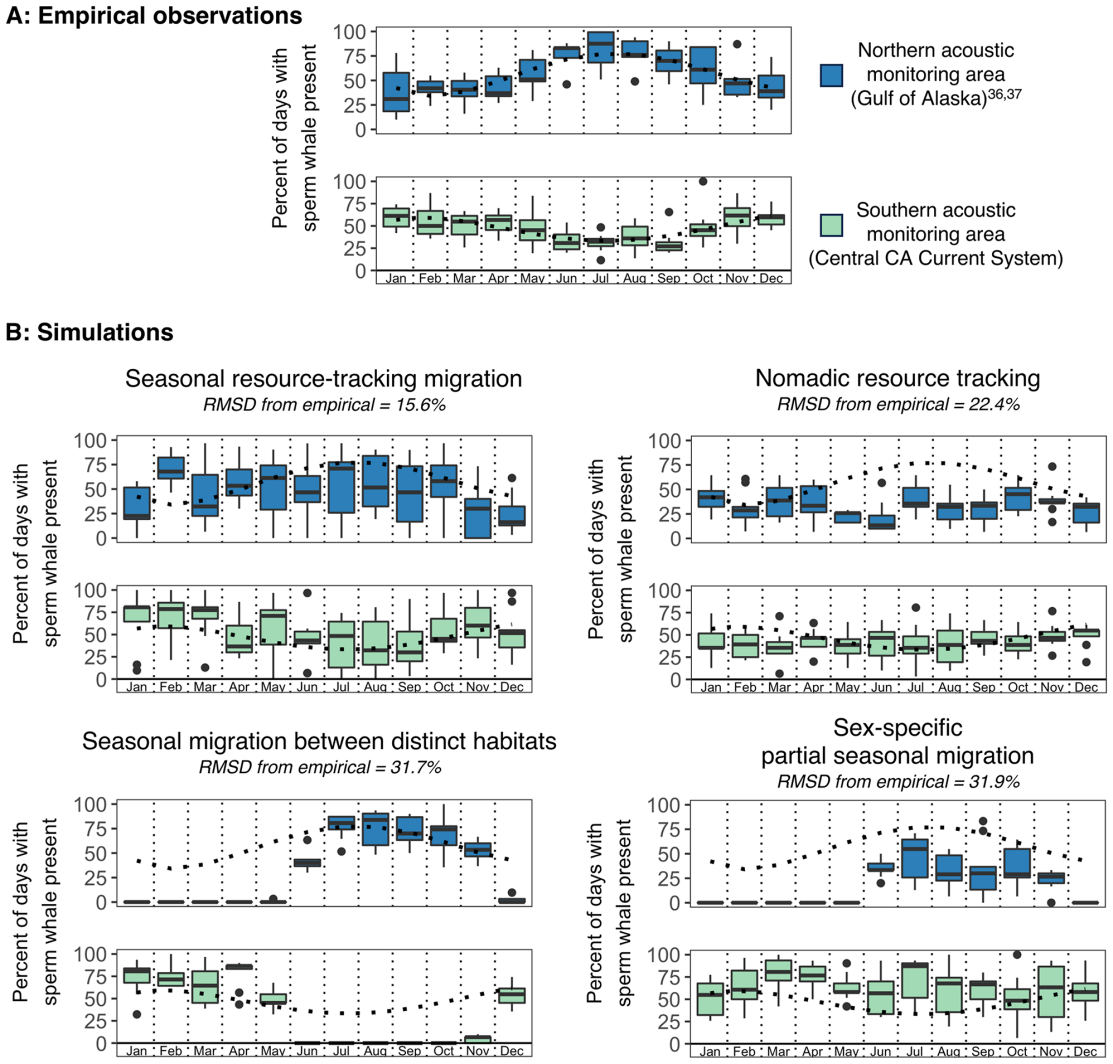
Comparison of empirical and simulated acoustic detection seasonality under hypothesized individual movement strategies. (**A**) Empirical acoustic detections from the Central California Current System (green; present study) and the Gulf of Alaska (blue; [36, 37]). Dotted curves represent a fourth-order polynomial fit to empirical monthly data from each recording site. (**B**) Acoustic detection at northern (blue) and southern (green) listening ranges for simulated agents following each of the hypothesized movement strategies. Boxplots show the median (center line), 25th -75th percentile (box), ± 1.5*IQR (whiskers), and outlying points of monthly acoustic detection over years 2–10 of each simulation. RMSD refers to the root-mean-square deviation of each simulation’s monthly mean acoustic detection results across both hydrophones relative to empirical observations. Empirical data fourth-order polynomial from (**A**) is overlaid on all simulated results

Simulations of individual-level movement yielded qualitatively and quantitatively distinct patterns in seasonal-latitudinal distribution (Fig. 2) and seasonal acoustic detection (Fig. 5), dependent on the movement strategy employed. The simulation of seasonal resource tracking individuals yielded year-round presence with moderate seasonality at both southern and northern listening ranges (Fig. 2A), peaking in the winter and summer for the southern and northern listening ranges, respectively (Fig. 5B). The seasonal patterns of acoustic detection arising from seasonal resource-tracking migration represented the only simulated results matching the defining qualities of empirically observed patterns: year-round presence with substantial and opposite seasonality at both higher and lower-latitude listening ranges (Fig. 5). Agents following nomadic resource tracking decision rules showed no seasonality in detection at northern or southern listening ranges (Fig. 5B), driven by similar winter and summer latitudinal distributions (Fig. 2B). Agents undertaking seasonal to-and-fro migrations between distinct habitats showed strong and opposite seasonality in latitudinal distribution (Fig. 2C). This simulation yielded a detection peak during winter and zero detections during summer at the southern listening range, while the northern listening range showed a summer peak in detections and zero detections during winter (Fig. 5B). Simulation of sex-specific partial seasonal migration resulted in strong detection seasonality at the northern listening range (high levels of detection in summer, zero detections in winter) and year-round detection with moderate seasonality at the southern listening range (Figs. 2D and 5B). Simulated acoustic detection patterns for seasonal resource-tracking migration were also quantitatively most similar to empirical acoustic detection, yielding a root-mean-square deviation among monthly means of only 15.6% (Fig. 5B). All other simulated movement strategies resulted in greater deviance from empirical observations (22.4% for nomadic resource tracking, 31.7% for seasonal to-and-fro migration between distinct habitats, 31.9% for sex-specific partial seasonal migration; Fig. 5B).

### Comparison to seasonally shifting oceanographic habitat

**Fig. 6:**
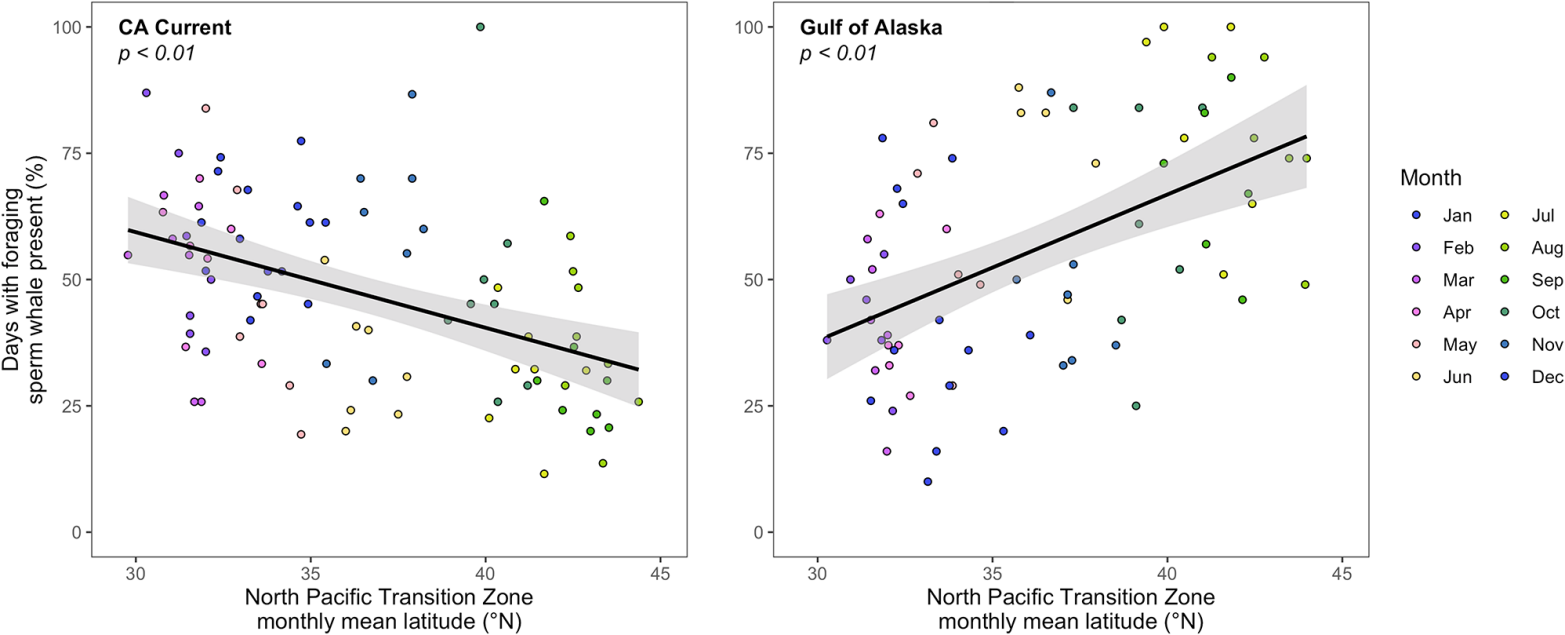
Foraging sperm whale presence follows oceanographic seasonality in the Northeast Pacific. Monthly empirically observed acoustic detection of foraging sperm whales in the Central California Current System and the Gulf of Alaska [36, 37] relative to the monthly mean latitude of the North Pacific Transition Zone. p-values reported for model II (ranged major axis; RMA) linear regression

Monthly percent presence of foraging sperm whales correlated with oceanographic seasonality in the Northeast Pacific Ocean (Fig. 6). The latitude of the North Pacific Transition Zone (NPTZ) was inversely correlated with foraging sperm whale presence in the CCCS (i.e., highest detection rate in the CCCS with NPTZ at its southern extent) and positively correlated with foraging sperm whale presence in the GoA (i.e., highest detection rate with NPTZ at its northern extent).

## Discussion

Animals’ movement strategies shape their ecology and their ability to respond to environmental perturbations. Moreover, these strategies offer a window into the spatiotemporal dynamics of the ecosystems they inhabit [1]. Our findings provide evidence for seasonal movements by a cryptic top predator in the deep ocean, the sperm whale. Below, we discuss several lines of evidence supporting this conclusion and consider how these findings advance understanding of seasonal movements in this population. More broadly, we discuss how these results advance knowledge of phenology in the poorly understood deep ocean ecosystems in which sperm whales forage.

The long-term acoustic detection results presented here indicate seasonality in the movements of foraging sperm whales, with greater frequency of echolocation click detection in California during winter (Fig. 3B; Figure S2), opposite the known summer peak of detection in the Gulf of Alaska [36–38] (Fig. 5A). Despite this opposite seasonality, foraging sperm whales are detected year-round in both locations. Based on several lines of evidence, we posit that these patterns indicate a seasonal migration in this population, likely driven by proximate resource tracking in an ecosystem with dampened seasonality. Seasonal resource-tracking migration is the only hypothesized movement strategy allowing for both year-round presence and significant seasonality in presence across latitudes (Figs. 2A and 5B), matching empirical estimates (Fig. 5A). Other hypothesized strategies yield either year-round presence (as in nomadism) or seasonality in acoustic detection across latitudes (as in full and sex-specific partial migration between distinct habitats), but do not match both of these key attributes of the empirical estimates (Fig. 5). Additionally, if sex-specific partial seasonal migration were occurring, we would expect the migratory demographic (previously hypothesized to be adult males [34, 43], with larger body sizes and higher inter-click-intervals (ICIs)) to drive seasonal patterns in the distribution of detected ICIs. Yet we do not observe any significant seasonal shifts in the monthly distribution of detected ICIs in California, instead detecting clicks consistent with female, juvenile, and adult male body sizes year-round (Fig. 4). We also find no relationship between monthly mean ICI and monthly percent presence (Figure S4), further indicating that the seasonal pattern observed in Fig. 3 is not driven by adult males alone. These results are consistent with long-term acoustic results from the GoA which also show year-round use of high latitudes by females, juveniles, and males [38]. This growing body of evidence from long-term, population-level observations via passive acoustics is inconsistent with the individual-sightings-based hypothesis of sex-specific latitudinal segregation, potentially arising from differences in the scale and persistence of observation [45, 46]. Climate change induced shifts in large-scale space use patterns of specific demographic groups could also influence these more recent observations of smaller individuals at higher latitudes. Even though significant uncertainty about the specific processes underlying these seasonal patterns remains, such continuous and detailed deep-sea acoustic observations provide useful insights toward enhancing our understanding of sperm whale behavior and phenology of the vast and opaque ecosystem they inhabit.

Despite seasonality in the frequency of foraging sperm whale presence, whales are still detected year-round across latitudes (Fig. 5A). This would be unexpected for a population migrating to track proximate resources in a strongly seasonal ecosystem (e.g., as in Northeast Pacific blue whales (*Balaenoptera musculus*) which forage and migrate in the epipelagic [18, 19]). However, one might expect subtle population-level seasonality of this nature for predators tracking resources in an ecosystem with a dampened seasonal cycle. There is growing evidence that deep sea ecosystems exhibit such dampened seasonality [26–28], resulting from an indirect relationship with seasonal solar variation mediated by organic matter falling from the directly seasonal surface ocean [23–25]. Seasonal resource-tracking migration in such an ecosystem can be considered an intermediate strategy between the seasonal resource-tracking movements previously studied in strongly seasonal ecosystems and the nomadic resource-tracking movements found in aseasonal ecosystems. Given that our simulation of nomadic resource tracking yielded the second-closest match to empirical observations (Fig. 5B), future work might use bio-logging and PAM in concert to test for individual-level variation along this continuum of nomadic to strongly seasonal resource tracking movements.

Our findings imply that sperm whales seasonally track a specific resource or resource-rich habitat in the Northeast Pacific. Ecosystem observations in sperm whales’ deep sea foraging habitat are sparse, preventing direct comparison between seasonal-latitudinal patterns of foraging sperm whale detection and deep-sea ecosystem observations. Whereas growing efforts to enhance deep sea observational capacity might allow more direct comparisons in the future, here we offer a preliminary comparison to the surface expression of the North Pacific Transition Zone, the dominant foraging habitat which numerous surface ocean predators track in this ocean basin [16, 50]. We tested whether sperm whales’ acoustically inferred seasonal-latitudinal movements track seasonal patterns in the latitude of the NPTZ. We find support for this hypothesis, with higher detection of foraging sperm whales at lower latitudes when the NPTZ is at its southern extent (and vice versa; Fig. 6). The considerable variation around this trend likely arises from the indirect link between surface biophysical processes (as measured via NPTZ latitude) and the behavior of a deep-sea top predator. Nevertheless, that this top predator of the deep ocean likely exhibits similar resource tracking behavior to that previously documented for surface ocean predators [16] suggests ecological links between surface and deep ocean processes and seasonality. Diel vertical migration of animals between the deep and surface ocean can vary seasonally in terms of depth distribution, total biomass, and carbon transport [27, 68–70]. In the Central California Current System specifically, total biomass throughout the meso- and epipelagic is at a minimum in spring and summer, rises in the fall, and remains elevated through the winter [27], allowing for greater transport of biomass between surface and deep waters during the seasons when foraging sperm whale detections peak in this region (Fig. 3B). It is important to note that we do not directly measure tracking of a forage resource here, and resource-tracking migrations can also include movements to track non-forage resources (e.g., predator-free habitat, favorable abiotic conditions, etc. [1, 71]), Still, the intensive energetic demands of raptorial feeding at sperm whales’ extreme body size [72] point to forage availability as a probable motivator of their movements in space and time.

While our findings shed light on the likely resource-tracking seasonal-scale movements of sperm whales in the Northeast Pacific, future work might explore the role of long-distance longitudinal movements. Northern elephant seals (*Mirounga angusirostris*) provide a valuable point of comparison in this regard, as these mesopelagic predators exhibit both longitudinal and latitudinal patterns in their seasonal movements [73, 74]. Indeed, sperm whales in the Pacific are also known to make long-distance longitudinal movements both within the Northeast Pacific and across the North Pacific more broadly [40], which could also contribute to observed seasonal patterns observed in the present study. Breeding phenology, hormonal and physiological changes associated with reproduction, and corresponding long-distance movements to lower-latitude calving grounds also must be considered. Yet sperm whales in the North Pacific exhibit seasonally diffuse breeding and a minority of the population bears young in any given year [75], suggesting that the seasonal patterns observed here result primarily from resource-tracking movements. Future research integrating population-level PAM observations with individual-level bio-logging observations would enable more detailed understanding of the drivers of sperm whales’ seasonal movements.

Seasonal resource-tracking migrations in terrestrial and epipelagic populations typically evolve as a strategy to maximize resource gain in dynamic, seasonal ecosystems [1, 4, 11]. Interannual variability around the average seasonal-latitudinal patterns exhibited by foraging sperm whales (Fig. 3) suggests that the cues driving their long-distance movements are not fixed seasonal cues (e.g., day length), thus affording flexibility to respond to environmental variation and change. Sperm whales were most often detected in the CCCS during 2016 (Fig. 3A), a year in which a persistent marine heatwave combined with a strong El Niño to drive widespread biological impacts in both the CCCS [76] and GoA [77]. By exhibiting a movement strategy driven by resource tracking rather than fidelity to a fixed foraging area or migratory schedule, sperm whales appear to respond flexibly to interannual variability in oceanographic conditions (Fig. 3A). Such flexibility is often characteristic of greater resilience to environmental perturbations [78] including marine heatwaves [79]. Understanding the individual and population-level outcomes of such flexibility in this sperm whale population remains an important and rich area for future study.

While the specific cues that enable these seasonal movements remain unclear, some combination of individual and social information is likely. As air-breathing predators, sperm whales spend significant time in surface waters subject to seasonal variability in solar irradiation, day length, and temperature. This provides a direct means of tracking progression of the seasons, perhaps enabling movements influenced by spatiotemporal memory similar to that observed in highly mobile epipelagic predators [19]. Because sperm whales echolocate to find prey, long-distance acoustic information on the foraging behavior of conspecifics might further direct this search, similar to the “mobile sensory networks” formed by echolocating bats [80]. Social learning of foraging and migration strategies could also play a role [81, 82], as sperm whales are highly social animals [34].

## Conclusions

Taken together, our findings suggest that growing evidence for seasonal processes in the deep ocean extend even to the seasonal movements of a top predator. This study underscores the need for additional research to understand phenology across trophic levels in light-limited deep pelagic ecosystems. A growing suite of technologies, including remotely operated vehicles, autonomous underwater vehicles, and continuous acoustic monitoring are providing an unprecedented opportunity to observe and understand deep ocean ecosystems [22, 28, 83]. Especially when integrated [28, 84], these tools can shed light on our murky understanding of seasonal processes and animals’ movement strategies in the deep sea. In turn, we can provide more precise scientific insight in support of spatiotemporally dynamic ecosystem management efforts which have to-date been used on land and in the surface ocean [85], but which may be possible and valuable in open and deep ocean ecosystems [86].

## Electronic supplementary material

Below is the link to the electronic supplementary material.

Supplementary Material 1

## Data Availability

Raw (256 kHz) and decimated (16 kHz) acoustic data from the MARS hydrophone are available at https://registry.opendata.aws/pacific-sound/. Code for processing acoustic data, analyzing sperm whale detections, and simulating individual-level movement strategies are available at 10.5281/zenodo.7860426.

## Abbreviations

BLED: Band Limited Energy Detector
CCCS: Central California Current System
GoA: Gulf of Alaska
ICI: Inter-Click-Interval
IDI: Inter-Detection-Interval
MARS: Monterey Accelerated Research System
NPTZ: North Pacific Transition Zone
