# Supplementary material for: Evidence for seasonal migration by a cryptic top predator of the deep sea
Source: Mov Ecol. 2024 Sep 24;12:65. doi: 10.1186/s40462-024-00500-x (PMC11421108; doi:10.1186/s40462-024-00500-x)
Supplement: Supplementary file 1 — Supplementary Material 1 [file 40462_2024_500_MOESM1_ESM.docx]

Supporting Information for:

**Acoustic evidence for seasonal migration by a cryptic top predator of the deep sea**

William K. Oestreich^a,^*, Kelly J. Benoit-Bird^a^, Briana Abrahms^b^, Tetyana Margolina^c^, John E. Joseph^c^, Yanwu Zhang^a^, Carlos A. Rueda^a^, John P. Ryan^a^

^a^ Monterey Bay Aquarium Research Institute, Moss Landing, CA 95039, USA

^b^ Center for Ecosystem Sentinels, Department of Biology, University of Washington, Seattle, WA 98195, USA

^c^ Naval Postgraduate School, Monterey, CA 93943, USA

*Corresponding author: William K. Oestreich

**Email:** [woestreich@mbari.org](mailto:woestreich@mbari.org)

**Supporting information on simulation of individual movement strategies**

The simulation methods used here follow methods introduced by Abrahms et al. (2017)^1^. Briefly, Abrahms et al. (2017)^1^ generated “computer-simulated idealized movement syndromes representing suites of correlated movement traits observed across taxa”. Their paper compared these idealized syndromes and their corresponding step length and turn angle distributions to 130 individual tracks from 13 vertebrate species exhibiting a range of movement strategies (migration, nomadism, territoriality, and central place foraging) in both marine and terrestrial ecosystems. These tracks ranged in duration from several months to multiple years, and were processed to standardized, 1-hour position sampling intervals given the importance of fix rate to step length and turn angle distributions. These authors found strong statistical evidence that their simple suite of seasonal movement rules, turn angle, and step length distributions for distinct movement strategies robustly represent the movement strategies in the diverse tracking dataset to which they were compared. These suites of distributions and movement rules therefore provide a useful set of dimensionless distributions representative of distinct vertebrate movement “syndromes”.

We applied these suites of distributions and movement rules to a dimensionless simulation domain which provides a simplified arena in which to explore the patterns of detection in two geographically-distant monitoring areas under hypothesized movement strategies. This domain is not meant to specifically represent the spatial dimensions of the North Pacific, but instead provides an arena for simplified hypothesis-testing roughly analogous to the geometry of our empirical sensors in the North Pacific. Agent step lengths, hydrophone listening ranges, and domain dimensions were scaled proportionally to give agents limited probability of acoustic detection even if present at the latitude of a listening range (i.e., listening ranges covered only a proportion of both the latitudinal and longitudinal dimensions). This approach allowed for realistic probabilities of acoustic detection for a large number of individual position-days (365,000 per simulation) without the extreme computational expense of simulating a number of agents comparable to the estimated population size of sperm whales in the eastern North Pacific (~2000^2^).

All distributions for step lengths and turn angle, as well as seasonality and other elements of movement rules, are summarized in Table S1. These simulations were implemented in a domain with longitude ranging from -2000 to 2000 and latitude ranging from 0 to 30000 (dimensionless units). Hydrophone “listening ranges” were positioned at (0, 5000) and (0, 25000).

*Nomadic resource tracking*

We simulated nomadic individuals using decision rules previously documented for nomads^1^: low probability (P=0.1) of behavioral state switching between active foraging and searching, small step lengths and uniformly-distributed turn angles during active foraging, and longer step lengths during searching with normally-distributed turn angles (around the initial direction after switching from foraging to searching).

*Seasonal resource-tracking migration*

We simulated movements to track resources with a shifting seasonal-latitudinal distribution using decision rules similar to those for nomadic resource tracking as described above, but with differences in movement behavior between times and locations of active foraging. Rather than searching in a single direction with turn angles normally-distributed around a randomly-selected initial search direction (as in nomads), agents in this simulation moved between active foraging periods by tracking resources with headings normally-distributed around due north and due south. The probability of northward-centered or southward-centered heading distributions during resource tracking varied seasonally to mimic seasonal shifts in latitudinal resource availability.

*Seasonal migration between distinct habitats*

We simulated migration between distinct habitats again using the decision rules documented by^1^: four months of foraging in a southern range (steps defined by uniform step length and turn angle distributions), two months of northward migration (longer step lengths and normal turn angle distribution centered on north), four months of foraging in a northern range (steps again defined by uniform step length and turn angle distributions), and finally two months of southward migration (longer step lengths and normal turn angle distribution centered on south).

*Sex-specific partial seasonal migration between distinct habitats*

We simulated sex-specific partial seasonal migration by assigning 50% of agents to a migratory (male) group and 50% of agents to a resident (female and juvenile) group. Migrants followed the decision rules described above for migration between distinct habitats; residents followed the decisions rules described above for nomadic resource tracking, but only in the southern portion of the simulation domain.

**Table S1.** Parameters for simulations of hypothesized individual-level movement strategies, derived from ^1^. † Precise migration onset and duration varies slightly by individual (sd = 5 days).

| **Simulated movement strategy** | **Behavioral states** | **Step length** | **Turn angle (°)** | **Behavioral transition probability** | **Seasonality** | **Demographic elements** |  |
| --- | --- | --- | --- | --- | --- | --- | --- |
| *Nomadic resource tracking* | Active foraging | gamma distribution (shape=2, rate=2) *100 | uniform distribution (min=0, max=360) | P = 0.1 (switch to searching) | N/A | N/A |  |
|  | Searching | uniform distribution (min=0, max=300) | If transitioning from forage to search: uniform distribution (min=0, max=360)  If continuing search: normal distribution (mean=preceding step angle, sd=60) | P = 0.1 (switch to active foraging) | N/A | N/A |  |
| *Seasonal resource-tracking migration* | Active foraging | gamma distribution (shape=2, rate=2) *100 | uniform distribution (min=0, max=360) | P = 0.1 (switch to searching) | N/A | N/A |  |
|  | Searching | uniform distribution (min=0, max=300) | normal distribution, either northward (mean=0, sd=10) or southward (mean=180, sd=10) | P = 0.1 (switch to active foraging) | probability of northward search varies seasonally (max of 0.8 in summer; min of 0.2 in winter) | N/A |  |
| **Simulated movement strategy** | **Behavioral states** | **Step length** | **Turn angle (°)** | **Behavioral transition probability** | **Seasonality** | **Demographic elements** |  |
| *Seasonal migration between distinct habitats* | Active foraging | gamma distribution (shape=2, rate=2) *100 | uniform distribution (min=0, max=360) | P = 0.1 (switch to searching) | During ~4-month periods between northward & southward migrations | N/A |  |
|  | Searching | uniform distribution (min=0, max=300) | If transitioning from forage to search: uniform distribution (min=0, max=360)  If continuing search: normal distribution (mean=preceding step angle, sd=60) | P = 0.1 (switch to active foraging) | During ~4-month periods between northward & southward migrations | N/A |  |
|  | Northward migration | constant (distance between habitats divided by ~2-month migration duration) | normal distribution (mean=0, sd=5) | N/A | ~2-month period in spring-summer † | N/A |  |
|  | Southward migration | constant (distance between habitats divided by ~2-month migration duration) | normal distribution (mean=180, sd=5) | N/A | ~2-month period in fall-winter † | N/A |  |
| **Simulated movement strategy** | | **Behavioral states** | **Step length** | **Turn angle (°)** | **Behavioral transition probability** | **Seasonality** | **Demographic elements** |
| *Sex-specific partial seasonal migration* | Active foraging | gamma distribution (shape=2, rate=2) *100 | uniform distribution (min=0, max=360) | P = 0.1 (switch to searching) | During ~4-month periods between northward & southward migrations | N/A |  |
|  | Searching | uniform distribution (min=0, max=300) | If transitioning from forage to search: uniform distribution (min=0, max=360)  If continuing search: normal distribution (mean=preceding step angle, sd=60) | P = 0.1 (switch to active foraging) | During ~4-month periods between northward & southward migrations | N/A |  |
|  | Northward migration | constant (distance between habitats divided by ~2-month migration duration) | normal distribution (mean=0, sd=5) | N/A | ~2-month period in spring-summer † | Assigned 50% of population undertakes migration |  |
|  | Southward migration | constant (distance between habitats divided by ~2-month migration duration) | normal distribution (mean=180, sd=5) | N/A | ~2-month period in fall-winter † | Assigned 50% of population undertakes migration |  |

**Supporting Information on automated detector performance assessment**

We assessed performance of the automated detector at daily resolution via manual comparison to 50 days (N) of acoustic data from the MARS dataset (1 day from each month of 2016, 2018, 2020 & 2022; and 2 days from known dates of sperm whale presence in Nov-Dec 2022). Detector performance was assessed for a range of required repeated potential click detections (from r = 3 to r = 10; Figure S1). Of these 50 days, manual assessment compared to the best performing automated detector (r = 6; Figure S1; Table S2) resulted in 26 true positives (TP), 22 true negatives (TN), 1 false positive (FP), and 1 false negative (FN). The following metrics were calculated to assess automated detector performance:

$$\boldsymbol{Precision=}\frac{\boldsymbol{TP}}{\boldsymbol{TP+FP}}$$

$$\boldsymbol{Recall=}\frac{\boldsymbol{TP}}{\boldsymbol{TP+FN}}$$

$$\boldsymbol{False positive rate=}\frac{\boldsymbol{FP}}{\boldsymbol{FP+TN}}$$

$$\boldsymbol{Specificity=}\frac{\boldsymbol{TN}}{\boldsymbol{TN+FP}}$$

$$\boldsymbol{Accuracy=}\frac{\boldsymbol{TP+TN}}{\boldsymbol{N}}$$

$$\boldsymbol{Balanced accuracy=}\frac{\boldsymbol{Recall+Specificity}}{\boldsymbol{2}}$$

*Table S2. Performance of automated daily acoustic processing relative to manual assessment dependent on number of near-constant inter-click interval repetitions required for detection. r = 6 (gray shading) yields best performance for the automated detector.*

| *r* | *Accuracy* | *Balanced accuracy* | *Precision* | *Recall* | *Specificity* | *False positive rate* | *TP* | *TN* | *FP* | *FN* |
| --- | --- | --- | --- | --- | --- | --- | --- | --- | --- | --- |
| *3* | *0.72* | *0.70* | *0.66* | *1.0* | *0.39* | *0.61* | *27* | *9* | *14* | *0* |
| *4* | *0.80* | *0.78* | *0.73* | *1.0* | *0.57* | *0.43* | *27* | *13* | *10* | *0* |
| *5* | *0.92* | *0.92* | *0.90* | *0.96* | *0.87* | *0.13* | *26* | *20* | *3* | *1* |
| *6* | *0.96* | *0.96* | *0.96* | *0.96* | *0.96* | *0.04* | *26* | *22* | *1* | *1* |
| *7* | *0.92* | *0.92* | *0.96* | *0.89* | *0.96* | *0.04* | *24* | *22* | *1* | *3* |
| *8* | *0.86* | *0.87* | *0.95* | *0.78* | *0.96* | *0.04* | *21* | *22* | *1* | *6* |
| *9* | *0.86* | *0.87* | *0.95* | *0.78* | *0.96* | *0.04* | *21* | *22* | *1* | *6* |
| *10* | *0.86* | *0.87* | *0.95* | *0.78* | *0.96* | *0.04* | *21* | *22* | *1* | *6* |


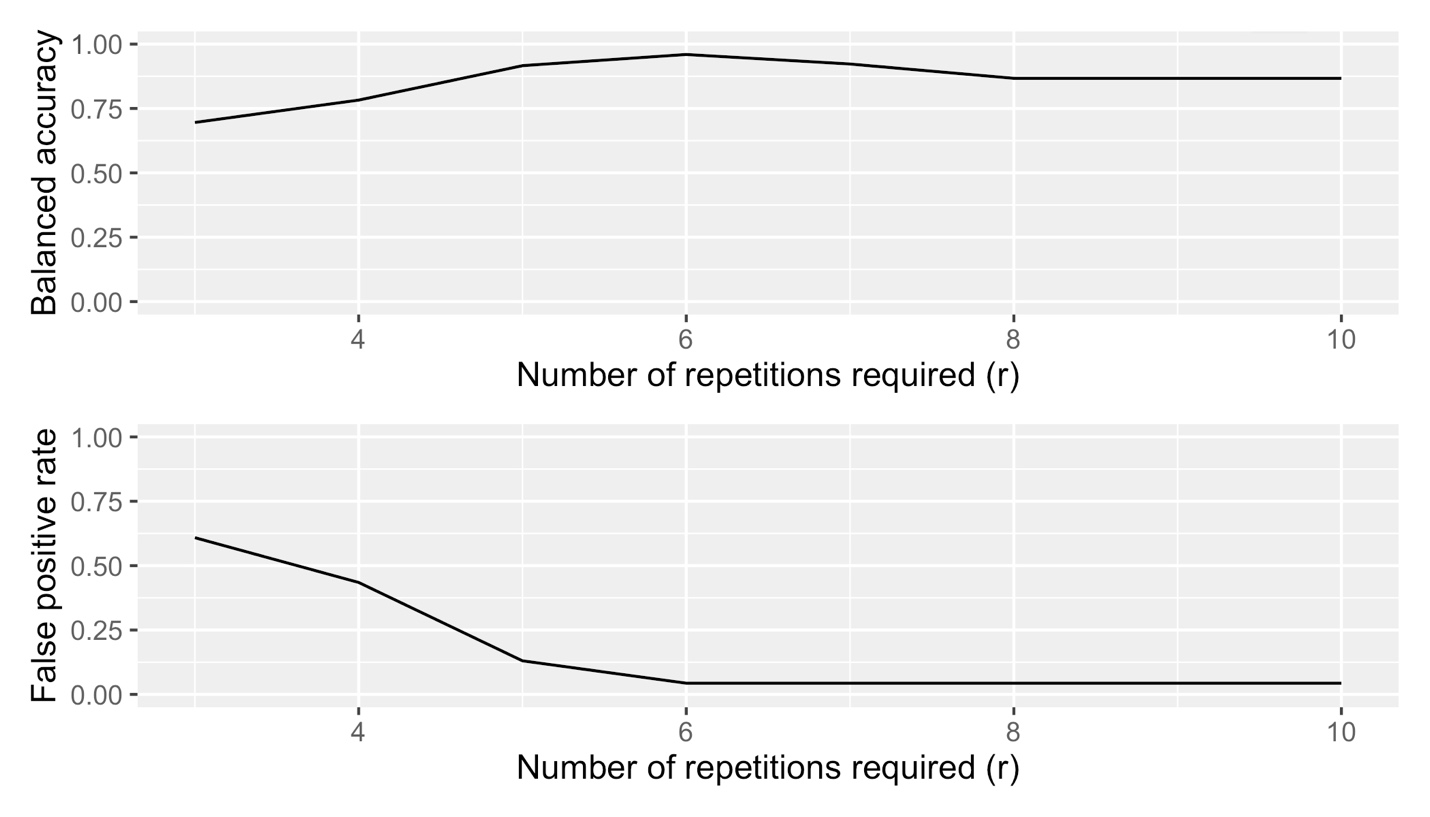


*Figure S1. Performance of automated daily acoustic processing relative to manual assessment. Requiring six repetitions of click detection at near-constant inter-click interval (r = 6) yields a daily balanced accuracy of 96% and daily false positive rate of 4%.*


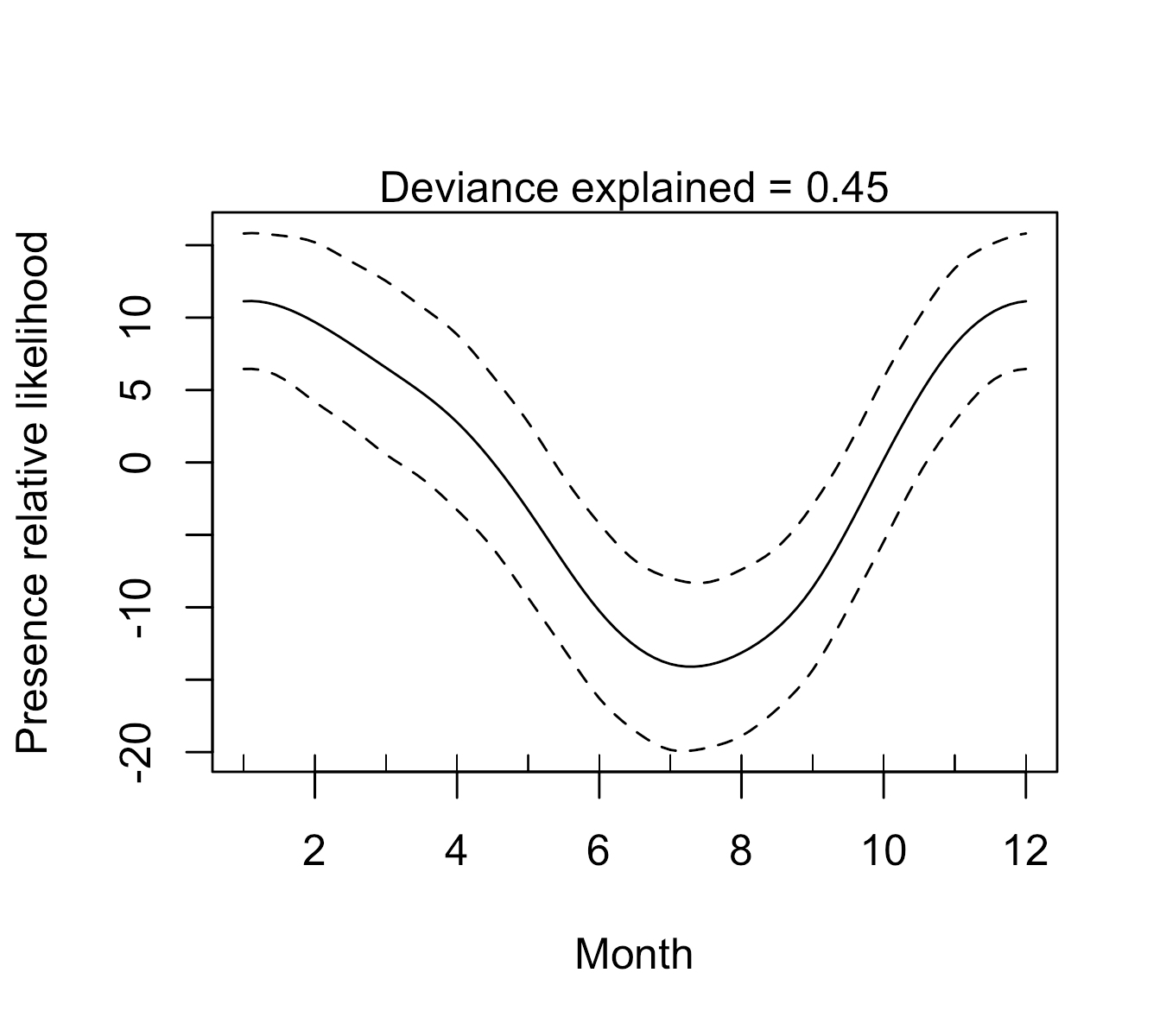


*Figure S2. Generalized additive model fit relationship for monthly foraging sperm whale presence (% of days) and month, with year nested as a random effect.*


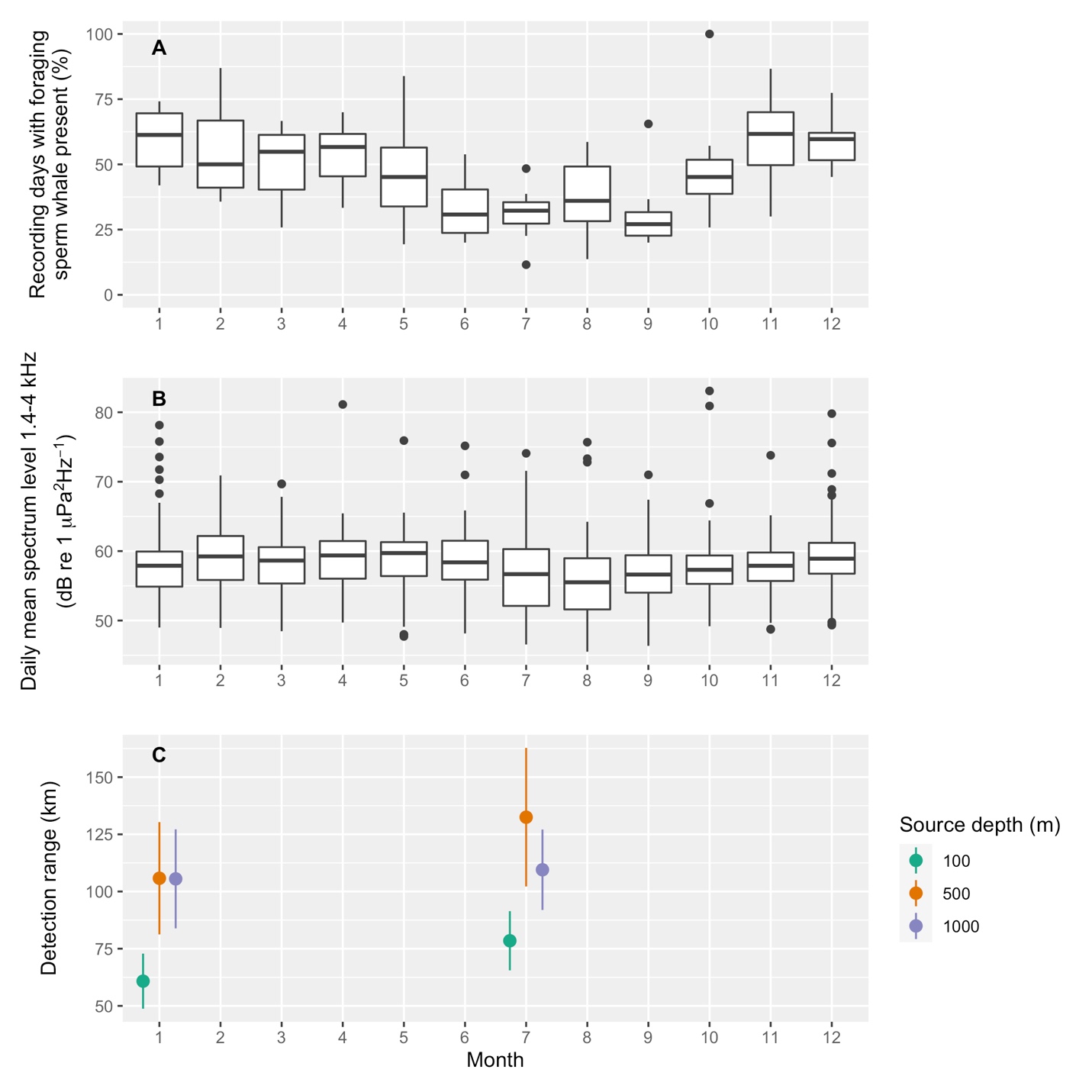


***Figure S3. Seasonal variation in listening conditions at MARS. (A)*** *Average annual cycle of echolocating sperm whale presence averaged over the full study period (Aug 2015 – Dec 2022), reproduced from Figure 3B in the main text.* ***(B)*** *Average annual cycle of ambient noise conditions at MARS in the frequency range (1.4-4kHz) targeted by the band limited energy detector employed to identify candidate sperm whale echolocation detections.* ***(C)*** *Estimated maximum detection range at MARS for sperm whale echolocation clicks produced at depths of 100, 500, and 1000m during the maximum (January) and minimum (July) months of foraging sperm whale presence. Points and lines represent the mean and standard deviation of 1-degree bearing ranges between 154-311° around MARS, representing the offshore area where 500m and 1000m source depth results are not limited by the shelf break (Figure 1B), and where sperm whales are most likely to be found. See Methods for information on modeling of acoustic propagation and detection range.*

**
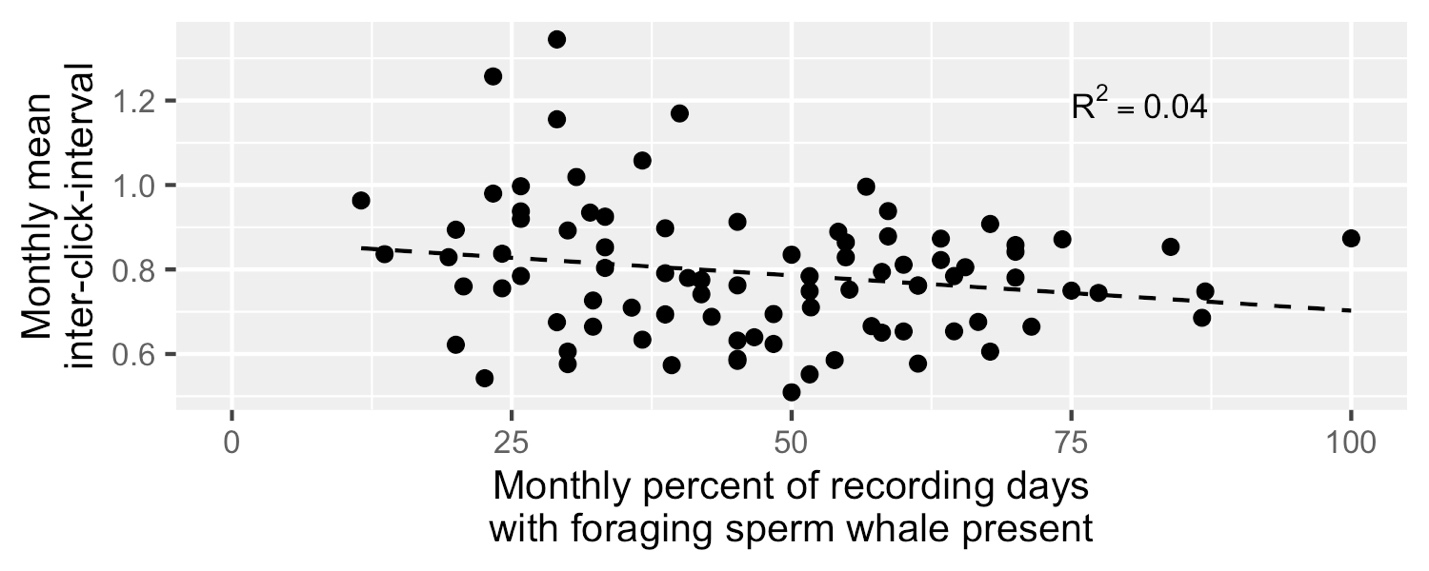
**

***Figure S4. Additional inter-click-interval (ICI) comparison to monthly foraging sperm whale presence.*** *Monthly mean ICI vs. monthly percent presence, indicating no significant relationship between these variables (p > 0.05).*

Table S3. Band limited energy detector parameters.

| BLED signal calculation | |
| --- | --- |
| Min. Frequency | 1.4 kHz |
| Max. Frequency | 4.0 kHz |
| Min. Duration | 8.125 ms |
| Max. Duration | 32.5 ms |
| Min. Separation | 32.5 ms |
| BLED noise calculation | |
| Block size | 2.0 s |
| Hop size | 0.5 s |
| Percentile | 20.0 |
| Signal-to-noise parameters | |
| Min. Occupancy | 70.0% |
| SNR Threshold | 5.0 dB |
| Spectrogram calculation | |
| Window | Hann |
| Window Size | 512 samples |
| Window Overlap | 95% |

**References**

1. Abrahms, B. *et al*. Suite of simple metrics reveals common movement syndromes across vertebrate taxa. *Mov. Ecol.* **5**, 12 (2017).
2. Moore, J. & Barlow, J. Improved abundance and trend estimates for sperm whales in the eastern North Pacific from Bayesian hierarchical modeling. *Endanger. Species Res.* **25**, 141–150 (2014).
